# Epstein-Barr Virus Independent Dysregulation of UBP43 Expression Alters Interferon-Stimulated Gene Expression in Burkitt Lymphoma

**DOI:** 10.1371/journal.pone.0006023

**Published:** 2009-06-24

**Authors:** Ingrid K. Ruf, Jennifer L. Houmani, Jeffery T. Sample

**Affiliations:** 1 Department of Molecular Biology and Biochemistry, University of California Irvine, Irvine, California, United States of America; 2 Department of Microbiology and Immunology, The Pennsylvania State University College of Medicine, Hershey, Pennsylvania, United States of America; Karolinska Institutet, Sweden

## Abstract

Epstein-Barr virus (EBV) persists as a life-long latent infection within memory B cells, but how EBV may circumvent the innate immune response within this virus reservoir is unclear. Recent studies suggest that the latency-associated non-coding RNAs of EBV may actually induce type I (antiviral) interferon production, raising the question of how EBV counters the negative consequences this is likely to have on viral persistence. We addressed this by examining the type I interferon response in Burkitt lymphoma (BL) cell lines, the only *in vitro* model of the restricted program of EBV latency-gene expression in persistently infected B cells *in vivo*. Importantly, we observed no effect of EBV on interferon alpha-induced signaling or evidence of type I interferon production, suggesting that EBV in this latent state is silent to the cell's innate antiviral surveillance. We did uncover, however, a defect in the negative feedback control of interferon signaling in a subpopulation of BL lines as was revealed by prolonged interferon-stimulated gene transcription consistent with sustained tyrosine phosphorylation on STAT1 and STAT2. This was due to inadequate induction of expression of the ubiquitin-specific protease UBP43, which removes the ubiquitin-like ISG15 polypeptide conjugated to proteins (ISGylation) in response to type I interferons. Results here are consistent with previous findings in genetically engineered *Ubp43^−/−^* murine cells that UBP43 down-regulates interferon signaling, independent of its ISG15 isopeptidase activity, by precluding the protein kinase JAK1 from the interferon receptor. This natural deficiency in UBP43 expression may therefore provide a useful model to further probe the biological roles of UBP43 and ISGylation.

## Introduction

Burkitt lymphoma (BL) is a B-cell tumor that arises as a consequence of chromosomal translocations that juxtapose the c-*MYC* proto-oncogene to Ig-gene enhancers, resulting in constitutive over-expression of c-MYC [1]. The endemic form of BL, furthermore, is highly associated (>90%) with latent infection by Epstein-Barr virus (EBV), though the precise contribution(s) of EBV to lymphomagenesis in this context is unclear. Cell lines derived from EBV-positive BL tumors, unlike EBV-immortalized B lymphoblastoid cell lines (LCLs), are not dependent on EBV for continued cell growth and proliferation, and maintain a restricted program of viral latent-infection gene expression known as Latency I, in which the only known viral protein expressed is the genome-maintenance protein, EBNA-1. In addition, the EBV small non-coding RNAs EBER-1 and EBER-2 are expressed, as are a family of alternatively spliced and potentially non-coding transcripts (*BARTs*) that are the precursors for a subgroup of the EBV miRNAs [2]–[4]. By contrast, LCLs (and some BL cell lines) support expression of the full complement of EBV latency-associated genes, i.e., the Latency III or Growth program.

Whereas Latency III represents viral gene expression that ensues for a limited time following infection of naïve B cells *in vivo*, virus-driven establishment of latent infection within memory B cells (the long-term reservoir of EBV) is believed to result in the complete cessation of EBV protein expression, i.e., the Latency program (reviewed in [5]). However, upon periodic replication of latently infected memory cells (presumably to promote self renewal), reactivation of EBNA-1 expression occurs to prevent loss of the episomal EBV genome during cell division [6]. BL cell lines that maintain the Latency I program, therefore, offer an *in vitro* model of EBV latency that is representative of persistent EBV infection in its normal B cell host.

Herein, we employed this model of restricted EBV latency to address whether the virus may likely interfere with the type I interferon (IFN) response in normal B cells persistently infected with EBV. This work was initially prompted by a report that EBER expression in BL cells confers resistance to IFN-α-induced apoptosis [7], but that in our hands this was not mediated through inhibition of the dsRNA-activated protein kinase (PKR) [8], as had been previously concluded [7]. More recently, work from others suggests that the EBER RNAs themselves induce the expression of type I IFNs through direct activation of RIG-I [9]. We reasoned, therefore, that as a consequence of IFN induction by the EBERs, EBV may have evolved a mechanism to counter antiviral responses and other influences of IFN on cellular processes counterproductive to long-term EBV persistence.

During Latency III, the LMP-1 protein of EBV inhibits the activation (Tyr phosphorylation) of the JAK kinase Tyk2 during IFN-α signaling [10]. We hypothesized, therefore, that a comparable inhibition of IFN-induced signaling may exist during Latency I (LMP-1 negative) to counter type I IFN production. However, contrary to our hypothesis, we report here that IFN-α-induced signaling, as measured by activating phosphorylation of STAT1 and STAT2 and the induction of IFN-stimulated gene (ISG) expression, is not affected by EBV in BL cells that maintain Latency I. We also did not observe evidence of IFN-α/β production by EBV-infected B cells. Instead, we uncovered an EBV-independent and previously unknown defect in the negative feedback regulation of IFN-α signaling. This was evidenced by sustained Tyr phosphorylation of STAT1 and STAT2 and DNA-binding by ISGF3 (the STAT1- and STAT2-containing complex responsible for induction of ISG transcription), resulting in prolonged ISG expression following IFN treatment. Further, we demonstrate that the basis for this is an inability to adequately express the ubiquitin-specific protease UBP43 (itself encoded by an ISG) that deconjugates the ISG15 protein moiety from ISGylated proteins [11], [12], and which has previously been shown to inhibit type I IFN signaling by blocking STAT phosphorylation and consequently ISG induction through its direct displacement of the kinase JAK1 from the IFN-α/β receptor [12], [13]. Thus, our data supports UBP43 as a primary negative regulator of IFN-α signaling. Finally, the naturally arising dysregulation of UBP43 expression described here, which to our knowledge has not been previously observed, occurs within a subset of BL cell lines derived from independent tumors, suggesting that it is not a random or isolated defect, but presumably one that confers an advantage to some BLs.

## Results

### Dysregulation of ISG Expression in BL

**Figure 1:**
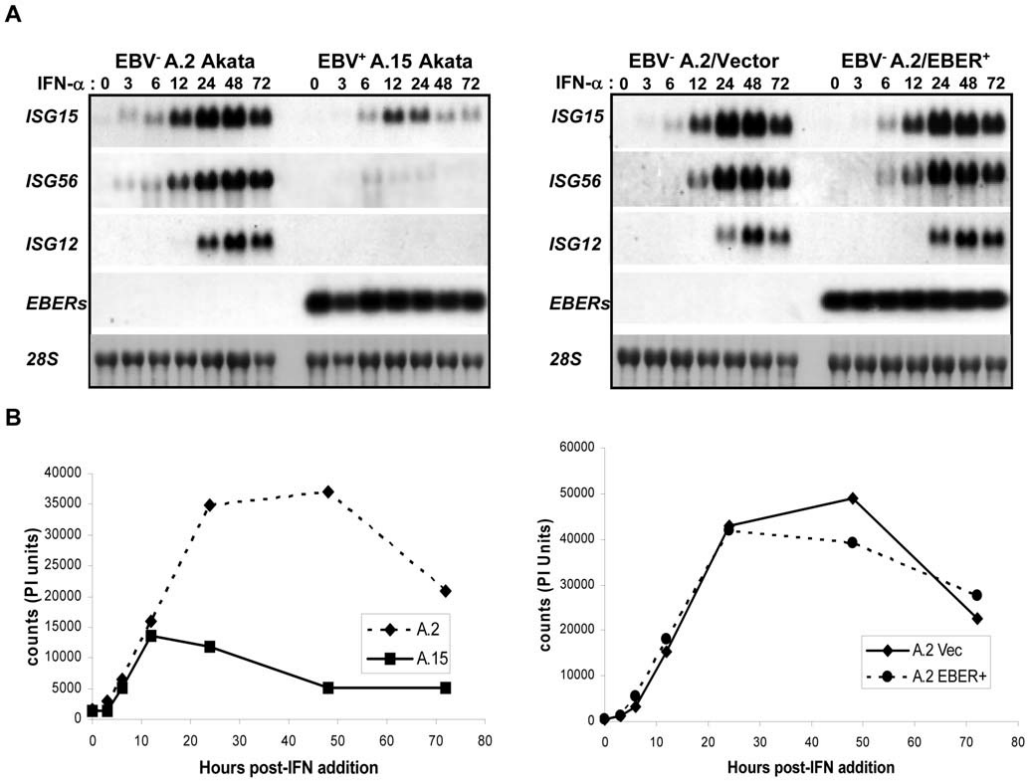
Differential induction of ISG mRNA in IFN-α-treated EBV-negative and EBV-positive Akata BL cell lines. A) Northern blot analysis of *ISG15*, *ISG56* and *ISG12* in EBV-negative (A.2) and EBV-positive (A.15) BL cells (left panel), and EBV-negative A.2 vector-control and EBER-expressing BL cells (right panel) following treatment with IFN-α (100 units/ml) for up to 72 h. Each lane contained 10 µg total cellular RNA. Analysis of the expression level of each RNA was determined by sequential probing of a single blot for each cell line. EBER expression was analyzed to confirm EBV status in A.15 cells and to determine EBER expression levels in EBV^−^/EBER^+^ cells. Levels of 28S rRNA were analyzed to ensure equivalent RNA loading and integrity. B) Quantification by phosphorimage analysis of *ISG15* mRNA levels from (A). Results are representative of two independent experiments.

To investigate the potential impact of the restricted program of EBV latency-gene expression on the type I IFN response, we first examined whether EBV, and in particular the EBER RNAs, influences IFN-α induction of cellular gene expression. The cell model we chose was a pair of EBV-positive and -negative cell lines derived from Akata BL cells that, for reasons that are unclear, can spontaneously lose the episomal EBV genome [14]. Loss of the EBV genome from these cells, which maintain a Latency I program, results in decreased tumorigenic potential and reduced resistance to apoptosis in response to IFN-α, both properties of EBV-positive Akata cells that are largely dependent on the EBERs [7], [8], [15]–[18]. Following addition of IFN-α (100 U/ml) to Akata-cell cultures, cell samples were removed at 3 to 72 h for extraction of total RNA, which was then analyzed by northern blot hybridization for the expression of *ISG15*, *ISG56* and *ISG12*. These ISGs were chosen based on an earlier microarray analysis that revealed constitutive (i.e., IFN-independent) expression of their mRNAs to be down-regulated in EBV-positive Akata cells relative to their EBV-negative counterparts (I.K.R. and J.T.S., unpublished observation). As shown in Fig. 1A (left panel), in the EBV-negative cells, IFN-α-induced ISG expression was not only substantially higher, it was also sustained for an additional 24–48 h, suggesting that EBV infection may suppress IFN-α signaling. To address this possibility, we repeated the analysis with the same EBV-negative cells (A.2) engineered to stably express the EBERs at physiologic levels, along with the EBV genome-maintenance protein, EBNA-1 [17]. However, as shown in Fig. 1A (right panel), the presence of these EBV gene products did not affect the intensity or duration of ISG expression in an otherwise EBV-negative background, suggesting that the different responses of these BL lines to IFN-α are not attributable to EBV (see also below). A quantification of *ISG15* expression in these cells is presented in Fig. 1B.

**Figure 2:**
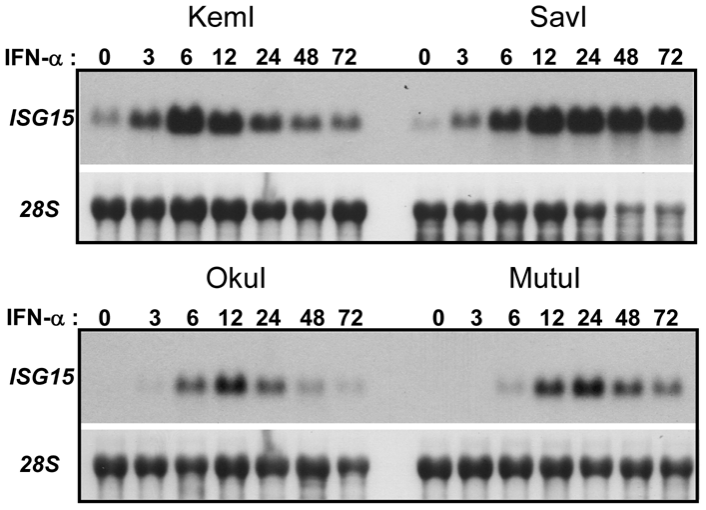
Differential IFN-α induction of *ISG15* mRNA expression among non-Akata EBV-positive BL cell lines. *ISG15* mRNA levels were analyzed from total RNA extracted from KemI, SavI, OkuI and MutuI BL cells that had been treated or not with IFN-α for 3–72 h. Data shown is representative of at least two independent experiments for each cell line.

To further assess a potential influence of EBV, we measured the response to IFN-α by three additional EBV-positive BL cell lines that maintain a Latency I program (KemI, SavI and MutuI), as well as one line (OkuI) that maintains a similar pattern of EBV latency-gene expression (Wp-restricted latency), but which also expresses the three EBNA-3 proteins (3A, 3B and 3C) [19]. As revealed by the analysis of *ISG15* mRNA expression (Fig. 2), KemI and OkuI cells were equivalent to EBV-positive Akata cells (as in Fig. 1), whereas SavI cells exhibited sustained expression of *ISG15* comparable to that observed for EBV-negative Akata cells; MutuI cells appeared to support an intermediate response to IFN-α. The data presented in Figs. 1 and 2, therefore, argue against an influence of EBV on the type I IFN induction of gene expression during the Latency I program of EBV infection, though we cannot rule-out the possibility that differences observed here were in fact due to the expression of a viral gene(s) that is not uniformly expressed in BL cells that otherwise maintain a Latency I program (i.e., other than EBNA-1, *EBERs*, *BARTs* and a subpopulation of the EBV miRNAs).

### Aberrant ISG Expression is Transcriptional

**Figure 3:**
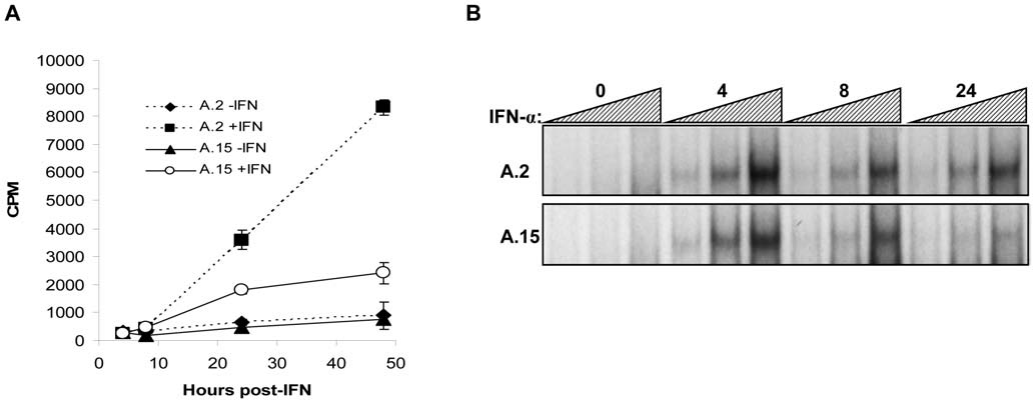
Elevated *ISG15* promoter and ISGF3 DNA-binding activities correspond to sustained *ISG* expression. A) A.2 and A.15 Akata BL cells were transfected in triplicate with 10 µg of an *ISG15* promoter-*hGH* reporter plasmid; after 14 h, IFN-α (100 units/ml) was added to half of the transfected cells and expression of hGH was determined in duplicate by radioimmunoassay at 4, 8, 24 and 48 h post-IFN-α addition. Results shown are representative of three independent experiments. B) EMSAs were performed with increasing amounts of protein (4, 8 or 16 µg) extracted from EBV-negative A.2 and EBV-positive A.15 Akata BL cells that had been treated with IFN-α for 0, 4, 8 or 24 h; the ^32^P-labeled double-stranded oligodeoxynucleotide probe contained the interferon-stimulated response element (ISRE) from the *ISG15* promoter.

We next considered the mechanistic basis for the observed differences in ISG expression. A comparative array analysis of IFN-α-induced gene expression in EBV-negative and -positive Akata cells had indicated that the majority of ISGs behaved like those analyzed in Fig. 1 (data not shown), suggesting that the differences we observed were due primarily to a common alteration in IFN-α-induced transcription. In support of this, we observed effects of IFN-α on *ISG15* promoter-driven reporter expression that paralleled the effects of IFN-α on endogenous ISG mRNA expression in EBV-negative and -positive Akata cells, i.e., induction of reporter expression was greater and sustained longer in the EBV-negative cells (Fig. 3A). Further, analysis by EMSA of the binding of ISGF3 (a complex of STAT1, STAT2 and IRF9) to a DNA probe containing the ISRE of the *ISG15* promoter indicated that IFN-α-induced binding to the probe was equivalent within extracts of EBV-negative (A.2) and EBV-positive (A.15) Akata cells for at least 4 h following addition of IFN-α. However, in contrast to EBV-negative cells in which binding was sustained for 24 h post-induction, ISGF3 binding was slightly reduced at 8 h and barely detectable at 24 h within extracts of EBV-positive cells (Fig. 3B). Collectively, these data indicated that differences in ISG expression in these BL cells are not due to a defect in the induction of gene expression by IFN-α, but rather an inability to sustain generation of ISGF3 (as in A.15 cells) or a block in the turnover of this transcriptionally active protein complex (as possible in A.2 cells). Given that the latter is considered part of the normal negative feedback restriction of the inductive phase of the type I IFN response (reviewed in [20]), we favored the conclusion that sustained expression of ISG transcription in a subset of BL lines, i.e., beyond several hours post induction, is an aberrant response.

### STAT Phosphorylation is Prolonged in Cells that Sustain ISG Expression

**Figure 4:**
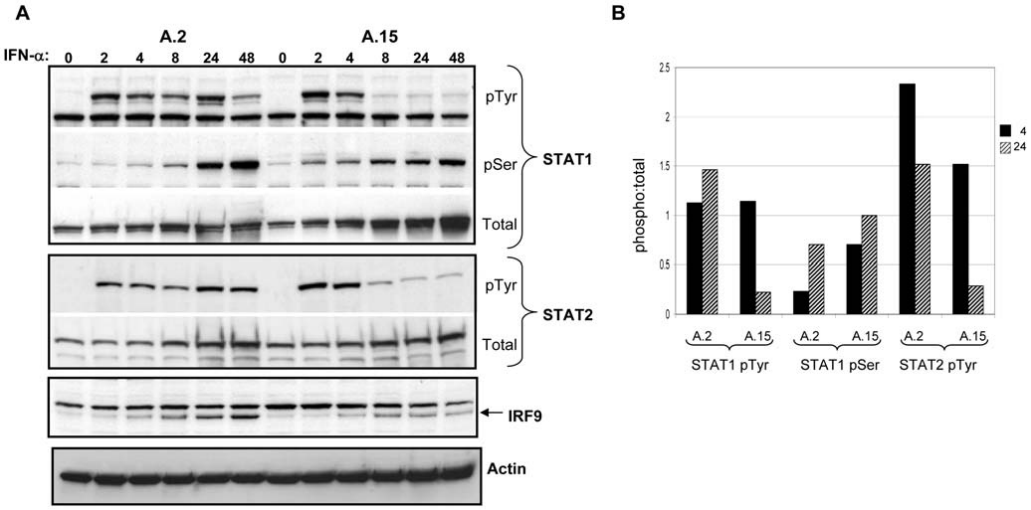
Tyrosine phosphorylation of STAT1 and STAT2 is prolonged in IFN-α-stimulated A.2 cells. A) Phosphorylation of STAT1 (pTyr^701^ and pSer^727^) and STAT2 (pTyr^689^) in response to IFN-α (100 U/ml) was monitored in EBV-negative A.2 and EBV-positive A.15 Akata BL cells by immunoblotting with phospho-specific antibodies. Blots were subsequently stripped and reprobed to detect total STAT1 and STAT2, as well as IRF9 and β-actin (loading control). B) Quantification of the ratio of phosphorylated STAT to total STAT (phospho:total) at 4 and 24 hours post-IFN addition on blots from (A).

Having determined that aberrant ISG expression is most likely due to sustained transcriptional activation by ISGF3, we next assessed the status of the components of this complex - STAT1, STAT2 and IRF9 - within IFN-α-treated cells that exhibited either normal or abnormal ISG expression. Consistent with the data presented in Figs. 1 and 3, within A.2 Akata cells (prolonged ISG expression) we observed sustained tyrosine phosphorylation of STAT1 (Tyr701) and STAT2 (Tyr689) throughout the 48-h time course of the experiment (Fig. 4). By contrast, within A.15 cells tyrosine phosphorylation of both proteins was dramatically reduced after 4 h, as expected. Phosphorylation of Ser727 on STAT1, which is not essential for STAT1 transcriptional activity [21], did not differ notably between A.2 and A.15 cells (Fig. 4). Expression of IRF9, the DNA-binding component of ISGF3 that does not require phosphorylation for activity, was equivalent in both cell lines prior to addition of IFN-α. Encoded by an ISG itself [22], IRF9 levels were lower in A.15 cells at later times post IFN-α treatment, as expected (Fig. 4). Thus, tyrosine phosphorylation or its absence on STATs 1 and 2 within A.2 and A.15 Akata cells at later times post IFN-α treatment was in good agreement with the detection or lack of ISGF3 binding to a classic ISRE in extracts of these cells, respectively (Fig. 3B), suggesting that sustained phosphorylation is the basis for prolonged ISG expression in A.2 cells.

**Figure 5:**
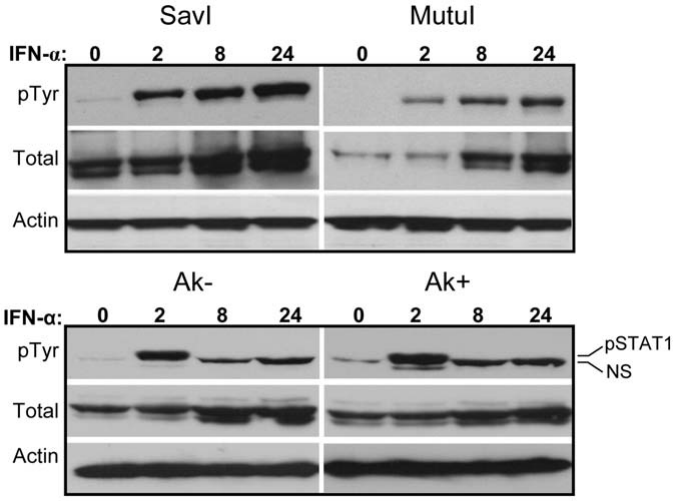
Duration of tyrosine phosphorylation of STAT1 correlates with sustained *ISG15* expression following IFN treatment. SavI and MutuI cells were treated with IFN-α for up to 24 h. Tyrosine phosphorylation of STAT1 was monitored by immunoblotting, as in Fig. 4, and then stripped and probed for total STAT1. A separate matched set of EBV-positive (Ak+) and EBV-negative (Ak−) Akata BL cells was also evaluated for STAT1 tyrosine phosphorylation and total STAT1 levels. Note: the Ak−/Ak+ blot was probed with a different lot of anti-phosho STAT1 antibody than used above or in Figs. 4 and 7. This antibody detected a nonspecific band (designated NS) with very similar mobility to pTyr^701^ STAT1. This band is unlikely to be a degradation product of STAT1 as we did not see this band using earlier lots of the antibody (see Fig. S1). The blots for β-actin served as protein loading controls.

To determine if this abnormal regulation of STAT phosphorylation is common to other BL cells that exhibited sustained ISG expression, we extended analysis of IFN-α-induced STAT1 Tyr701 phosphorylation to MutuI and SavI, which exhibited intermediate and sustained ISG responses, respectively, relative to A.2 Akata cells. We also assessed two additional and independently derived pair of subclonal EBV-negative (Ak− and 2A8−) and EBV-positive (Ak+ and 2A8+) Akata cell lines. As shown in Figs. 5 and S1, phosphorylation of STAT1 in MutuI and SavI BL cells was indeed sustained for at least 24 h, consistent with the prolonged ISG expression observed previously in these cells (see Fig. 2). Interestingly, unlike A.2 cells, Ak− and 2A8− EBV-negative Akata cells (and their EBV-positive counterparts) did not exhibit prolonged STAT1 phosphorylation, providing further evidence that the type I IFN response is not targeted by EBV during the Latency I program. Further, KemI cells, which exhibited a normal course of *ISG15* expression (Fig. 2), also exhibited normal kinetics of STAT1 phosphorylation, as expected (Fig. S1).

### Sustained STAT1 Phosphorylation is Due to Inadequate UBP43 Expression

Physiologic down-regulation of IFN-induced gene expression can occur through several mechanisms. Among these, two that we are aware of have a direct negative influence on the phosphorylation of STAT proteins by type I IFNs, as observed here. The first is the well established abrogation of STAT1 phosphorylation by suppressor of cytokine signaling 1 (SOCS1), itself an ISG protein [23]. We initially asked, therefore, whether a deficiency in SOCS1 expression might be responsible for sustained phosphorylation of STAT proteins in cells that support prolonged ISG expression. However, we observed no difference in constitutive SOCS1 expression, and IFN-α-induced expression of SOCS1 was actually slightly lower in A.15 relative to A.2 cells (data not shown), arguing against involvement of SOCS1 (assuming SOCS1 is biochemically active in A.2 and similar BL cell lines).

**Figure 6:**
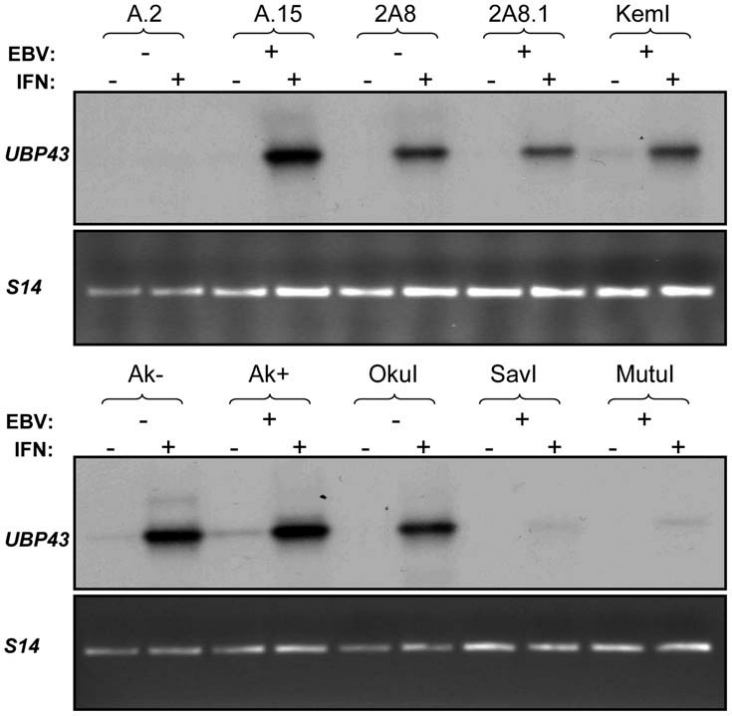
Reduction of *UBP43* mRNA corresponds to sustained ISG expression in BL cells. Total RNA was isolated from the indicated cell lines pre- and post-IFN-α treatment for 24 h and subjected to RT-PCR with *UBP43*- and ribosomal protein gene *S14*-specific primers. Amplified *UBP43* and *S14* (control for RNA integrity and amplification efficiency) cDNAs were detected by Southern blotting and ethidium bromide staining, respectively. Equivalent results were obtained in multiple independent experiments.

The second mechanism is the recently described inhibition of STAT1 phosphorylation by UBP43 (the product of *ISG43*) through its inhibition of JAK1 interaction with the IFNAR2 subunit of the type I IFN receptor [13]. Serendipitously, *ISG43* was one of the ISGs we initially selected to monitor in our assessment of IFN-α-induced transcription. Interestingly, unlike the results shown in Fig. 1 for *ISG15*, *ISG56* and *ISG12*, we observed little or no IFN-α induction of the *ISG43* mRNA by northern blotting in A.2 Akata cells (which exhibited high and prolonged ISG expression), whereas induction of *ISG43* expression was easily detectable in A.15 cells (data not shown). Thus, lack of an *ISG43*-encoded function might be responsible for the inability to efficiently terminate IFN-α-induced transcription in a subset of BL cells. To address the potential role of UBP43, we first assessed induction of its mRNA by IFN-α in our full panel of BL cell lines. As shown in Fig. 6, the induction of UBP43 mRNA expression was very low in all cell lines analyzed that exhibit prolonged ISG expression (A.2, SavI and MutuI), while notably higher in the lines that support normal kinetics of ISG expression. The latter group also included an additional EBV-negative Akata line (2A8) and its EBV-reinfected counterpart (2A8.1), providing further evidence that differences in IFN-α-induced gene expression (including UBP43) are EBV-independent.

**Figure 7:**
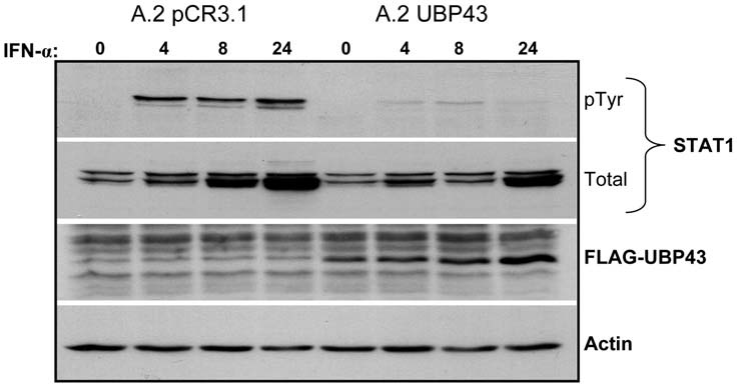
Constitutive expression of UBP43 reduces the induction and duration of IFN-α-induced tyrosine phosphorylation of STAT1. A.2 pCR3.1 (vector control) and A.2 UBP43 cells were treated with IFN-α (100 U/ml) for up to 24 h. Phosphorylated (pTyr) and total STAT1, FLAG-tagged UBP43 and β-actin (loading control) were detected by immunoblotting. Data shown is representative of three independent experiments.

Having demonstrated a direct correlation between the level of UBP43 mRNA induction and extent of ISG expression, we next tested whether exogenous expression of UBP43 could suppress IFN-α-induced phosphorylation of STAT1 in BL cells (A.2) in which phosphorylation is otherwise sustained. To do this, we stably expressed FLAG-tagged UBP43 in A.2 Akata cells, and compared IFN-α-induced phosphorylation of STAT1 on Tyr701 in these cells to a vector-only control line. As shown in Fig. 7, UBP43 expression caused a dramatic reduction in the amount of phosphorylated relative to total STAT1, consistent with the previous reports of UBP43-mediated inhibition of STAT1 phosphorylation and sustained STAT1 phosphorylation in *Ubp43^−/−^* cells [12], [24]. We conclude, therefore, that the inability to efficiently terminate IFN-α-induced signaling in a subset of BLs is due to insufficient expression of UBP43.

## Discussion

Here we report that a subpopulation of B-cell lines derived from BL tumors exhibit abnormally long IFN-α induction of gene expression that is independent of EBV infection. Although we cannot formally rule out the possibility that the observed differences in ISG induction among EBV-positive BL cells might be attributable to expression of viral genes not uniformly expressed in all EBV-positive BL cells, our finding that some EBV-negative BL cells also exhibit these differences in ISG induction supports the idea that this is indeed a virus-independent effect. This atypical response to IFN-α induction is associated with prolonged tyrosine phosphorylation on STAT1 and STAT2 and, consistent with the presence of these activated STAT proteins, extended DNA-binding activity of the ISGF3 complex, as well as higher and sustained expression of an ISRE-driven reporter gene. An exception to the generally sustained ISG expression in these BL cells was *ISG43*, which was minimally induced in all lines supporting prolonged ISG expression. This, and reports that the ubiquitin-specific protease UBP43 (alternatively USP18) encoded by *ISG43* can block type I IFN-induced phosphorylation of Tyr701 on STAT1 and that ISG expression is prolonged in *ubp43^−/−^* murine embryonic fibroblast cells [12], led us to examine whether inadequate induction of UBP43 expression is the basis for our observations. Indeed, stable expression of UBP43 in our variant BL cells substantially reduced IFN-α activation of STAT1. Thus, we conclude that a defect in the IFN-inducible expression of UBP43 results in a significant delay of the negative feedback control of type I IFN signaling in these tumor cells, irrespective of their EBV status.

UBP43 removes the ubiquitin-like protein encoded by *ISG15* that is conjugated to proteins in a process known as ISGylation [11], [25]. The biological importance of IFN-induced ISGylation is not fully understood, as it is not required for type I IFN signal transduction, ISG expression, or IFN-mediated antiviral functions [13], [26]–[28]. The isopeptidase activity of UBP43 responsible for deconjugation of ISG15ylated proteins, however, is not required for the inhibition of STAT1 phosphorylation, which appears to be mediated instead through direct inhibition by UBP43 of the interaction of JAK1 with the type I IFN receptor [13]. The defect in the UBP43-mediated negative feedback control of IFN signaling that we have uncovered is in the IFN-α-induced expression of UBP43, either in the transcriptional activation of the UBP43 gene by IFN-α, or in a co- or post-transcriptional event that results in a specific reduction in UBP43 mRNA production. On the surface, a defect in IFN-induced transcription would appear less likely given that *ISG43* is an immediate-early ISG [29], [30] and that there is not an apparent defect in classic ISGF3 activation of ISG transcription (e.g., as that of *ISG15*). However, we cannot rule out loss of contribution by an additional factor(s) needed for appropriate IFN-induced transcription of *ISG43*, but which is not required for the expression of the ISG family in general. Currently, however, little is known about the specific regulation of UBP43 expression at the transcriptional level.

An obvious question raised by our findings is what advantage is conferred to a cell, normal or transformed, from a sustained response to type I IFNs? While UBP43 deficiency results in extended expression of ISGs, leading to increased resistance to viral and some bacterial infections [11], [24], [28], [31], [32], it is difficult to rationalize heightened resistance to potential infection as the primary pressure to dysregulate UBP43 expression. Alternatively, suboptimal expression of UBP43, and the resulting delay in the negative feedback regulation of IFN signaling, may promote a beneficial IFN-dependent function not necessarily related to the antiviral response. Of the several hundred known ISGs, clearly not all are involved in mediating resistance to infections [22]. Further, UBP43 is constitutively expressed in liver, cells of the monocytic lineage and within fetal spleen [29], [33], [34], and in *Ubp43^−/−^* mice the complete lack of UBP43 expression is associated with elevated ISG15-conjugation and cellular necrosis in brain that results in severe neurological disorders [12], [27], [35], [36]. Thus, UBP43 appears to also contribute to a homeostatic (i.e., IFN-independent) regulation of ISGylation, though the *ISG15* gene does not appear to be required for these detrimental effects [13], [26], [27]. Thus, given the generally detrimental effect that prolonged IFN signaling would have on a cell, and the negative consequences UBP43 deficiency has in some tissues, it would seem that dysregulation of UBP43 as reported here would have greater relevance in the setting of oncogenesis and tumor maintenance, as transformed cells are characteristically resistant to at least some of the negative properties of IFN, such as antiproliferative and proapoptotic effects. Though few, there have been reports of UBP43 function related to oncogenesis: *1)* A UBP43-mediated block in cytokine-induced terminal differentiation of the myeloid cell line M1 [33]; *2)* A potential anti-neoplastic influence in acute promyelocytic leukemia [33], [34]; and *3)* Resistance to BCR-ABL induction of a chronic myeloid leukemia-like myeloproliferative disease in *Ubp43^−/−^* mice [37]. Of these, only the second report is consistent with a potentially pro-oncogenic consequence of UBP43 deficiency. However, this specific example would appear to have little relevance to BL.

Our original intent for these studies was to determine whether the restricted latency program of EBV interferes with the type I IFN response, the impetus for which were findings from our group and others that the EBER RNAs of EBV inhibit IFN-α-induced apoptosis [7], [8], and reports from Takada and colleagues that the EBERs induce expression of IFN-α/β through direct activation of RIG-I [9]. Specifically, the latter suggested to us that, given the potent antiviral properties of the type I IFNs, it is quite possible that EBV negatively targets the IFN-α-mediated signaling that would be initiated as a consequence of EBER induction of type I IFN expression. However, in contrast to the inhibition of type I IFN signaling by the LMP-1 protein of EBV during the growth or Latency III program [10], we found no evidence of such an effect in this cell model of restricted EBV latency. Interestingly, we also saw no indication of significant IFN-α/β production either from latently infected BL cells, or by EBV-negative BL cells that stably express the EBER RNAs (in addition to EBNA-1) at physiologic levels. Specifically, we did not detect tyrosine phosphorylation of either STAT1 or STAT2 in the absence of added IFN (Figs. 4 and 5), and consistent with this, little or no ISG mRNA expression (Figs. 1, 2 and 6). Note that a low level of *ISG15* mRNA detected in the absence of IFN-α (as in KemI and SavI cells, Fig. 2) is consistent with basal expression of *ISG15* and a subset of other ISGs [22]. Further, and consistent with the lack of detectable STAT1/2 phosphorylation in these BL cells, we have been consistently unable to directly detect IFN-α production (by ELISA) from our panel of BL cell lines, including EBV-negative Akata cells that stably express both EBERs (data not shown).

This apparent discrepancy with the earlier report may be due to the different experimental systems employed. Notably, the previous studies implicating EBER-induction of type I IFN production through direct activation of RIG-I relied on transfection (by electroporation) for the expression of EBERs and/or a GFP-tagged version of RIG-I (RIG-I/GFP) to show an effect [9]. This may have resulted in a functional interaction between the otherwise nuclear EBERs [38] and cytoplasmic RIG-I that normally would not occur during EBV latency. The activation of endogenous RIG-I (as evidenced by type I IFN expression) was not assessed in BL cells stably expressing the EBERs either naturally from the EBV genome, or by stable expression from an exogenously introduced vector in EBV-negative BL cells. Further, upon infection of EBV-negative BL cells, induction of IFN-β RNA expression could only be detected in cells stably overexpressing RIG-I/GFP [9]. Thus, it would appear that induction of type I IFNs by the EBERs is not a normal function of these noncoding RNAs, at least within the context of BL cells.

In conclusion, we have uncovered a defect in the negative feedback control of type I IFN signaling by the ISG15-specific isopeptidase UBP43 within BL cells. This is unlikely to represent a common cellular adaptation to enhance an IFN-induced antiviral response to EBV or other infectious agents, as it does not occur uniformly among EBV-positive BL cell lines, and such an antiviral response would likely be inconsistent with the high incidence of EBV infection associated with endemic BL. Further, the observation that the underlying mechanism of prolonged IFN signaling is also evident in several cell lines derived from independent tumors suggests that this is not a random isolated event, but rather that dysregulation of UBP43 expression confers relatively frequently an advantage to some tumor cells, though what this advantage may be is currently unclear. However, because UBP43 is not constitutively expressed in BL cells, we presume that any such advantage may lie in the effect that IFN-induced ISG15ylation has on protein function. While earlier studies have relied on gene knock-out technology to elucidate the biochemical functions of UBP43 [13], [26], [27], [35], this is the first instance that we are aware of in which a natural deficiency in UBP43 expression was observed. BL lines that exhibit a defect in the type I IFN-induced expression of UBP43 may therefore provide a useful model to further probe the biological roles of UBP43 and ISGylation and their contributions to cell biology.

## Materials and Methods

### Cell Culture and Plasmids

Cells were maintained in RPMI 1640 medium containing 2 mM L-glutamine (Mediatech) and 10% defined fetal bovine serum (HyClone). Akata A.2 and A.15 cell lines are EBV-negative and -positive derivatives, respectively, of the parental Akata BL cell line. A.2.EBER and A.2.Vector are A.2 Akata cells that stably express physiologic levels of EBER-1 and EBER-2, or which contain an empty EBER expression vector, respectively [17]. Cell line 2A8 is an independently-derived EBV-negative Akata clone (gift of J.W. Sixbey). Isolation and characterization of all Akata cell lines, as well as re-infection of 2A8 with EBV to generate 2A8.1, has been previously described [17], [18]. Akata Ak^−^ (EBV-negative) and Ak^+^ (EBV-positive) cell lines were kindly provided by K. Takada. KemI, SavI and MutuI are EBV-positive BL cell lines that maintain a Latency I program of EBV gene expression; OkuI BL cells are similar, but also express EBNAs 3A, 3B and 3C [19]. To establish an A.2 cell line that constitutively expressed UBP43, the UBP43 open reading frame was generated by PCR from a sequence-verified full-length cDNA purchased from Open Biosystems. PCR primers were designed to incorporate an N-terminal FLAG epitope as well as restriction sites for cloning. Primers used were: 5′-ACGTGGATCCGCCACCATGGATTACAAGGATGACGACGATAAGAGCAAGGCGTTTGGGCTCCTG-3′ and 5′-GATCCTCGAGTAGAAGACTCCGTAGATCCAG-3′. Following amplification, PCR products were digested with *Bam*HI and *Xho*I, and cloned into *Bam*HI- and *Xho*I-digested pCR3.1 (Invitrogen) to generate an expression vector encoding FLAG-UBP43. Ten micrograms of FLAG-UBP43 or pCR3.1 (to generate vector-control lines) was used to transfect 8×10^6^ A.2 cells by electroporation as described previously [39]. Stable transfectants were selected in 200 µg of G418 per ml. Following selection and expansion of cells, clones expressing UBP43 were identified by immunoblotting with anti-FLAG antibody (M2; Sigma).

### Analysis of RNA Expression

For analysis of IFN-induced RNA levels, cells were treated with hu-IFN-α-A (PBL Biomedical Laboratories) as described in the text. Total cellular RNA was isolated from 10^7^ cells with RNA-Bee as recommended by the manufacturer (Tel-Test), followed by extraction with an equal volume of phenol-chloroform and then chloroform prior to ethanol precipitation. For RNA (northern) blot hybridization, 10 µg of RNA was fractionated by electrophoresis in a 1.2% agarose-2.2 M formaldehyde gel, followed by transfer to a GeneScreen Plus membrane (PerkinElmer Life Sciences, Inc.). RNA blots were subjected to hybridization to ^32^P-labeled (by nick translation) DNA probes, washed, processed by autoradiography and quantified by PhosphorImage analysis (Molecular Dynamics). Blots were stripped and rehybridized to a probe specific for 28S rRNA to control for differences in RNA loading. For analysis of UBP43 mRNA levels by reverse transcription (RT)-PCR, 2.5 µg of total RNA isolated from untreated or IFN-α-treated cells was reverse-transcribed using the iScript cDNA synthesis kit (BioRad). Control reactions lacking reverse transcriptase were run in parallel. One-tenth of each cDNA reaction was then amplified using either *UBP43*- or ribosomal protein gene *S14*-specific primers: UBP43, 5′-AGCAAGGCGTTTGGGCTCCTG-3′ and 5′-GATCCTCGAGTAGAAGACTCCGTAGATCCAG-3′; S14, 5′-GGCAGACCGAGATGAATCCTCA-3′ and 5′-CAGGTCCAGGGGTCTTGGTCC-3′. Amplification was for 25 cycles at 95°C for 2 min, 58°C (*UBP43*) or 55°C (*S14*) for 1 min, and 72°C for 2.5 min (*UBP43*) or 1.5 min (*S14*); after the final cycle of amplification, samples were maintained at 72°C for 5 min. Following amplification, one-tenth of each product was electrophoresed in a 1.5% agarose gel, transferred to GeneScreen Plus membrane and processed by standard Southern blot hybridization techniques to detect UBP43 cDNA.

### DNA Transfection and Reporter Assay

Cells were maintained in roller bottle cultures for at least two feedings prior to transfection by electroporation, as previously described [39]. Briefly, 8×10^6^ cells were transfected in triplicate with 10 µg of pISG15-hGH reporter plasmid. pISG15-hGH was created by PCR amplification of bases −125 to +50 of *ISG15* from human genomic DNA (Promega) utilizing the following primers: ISG15a: 5′-GGGCATGCCTCGGGAAAGGG-3′ and ISG15b: 5′-GGCACGAGCTCCTGTACTGG-3′. The resulting fragment was blunt-end ligated into the *Bam*HI site of the human growth hormone (hGH) reporter plasmid pΦGH (Nichols Institute). At 14 h post-transfection, hu-IFN-α-A (PBL Biomedical Laboratories) was added to cell cultures to a final concentration of 100 U/ml. The level of hGH in the culture medium was determined by radioimmunoassay (Nichols Institute) in duplicate at various intervals following addition of IFN-α.

### Electrophoretic Mobility Shift Assay (EMSA)

For preparation of whole-cell extracts, 2×10^7^ cells either untreated or treated with IFN-α (100 U/ml) for 4, 8, or 24 h were washed in phosphate-buffered saline (PBS) and resuspended in 200 µl extraction buffer (20 mM HEPES-KOH [pH 7.9], 450 mM NaCl, 0.2 mM EDTA, 0.5 mM DTT, 25% glycerol, 1 mM sodium orthovanadate, 1 mM phenylmethylsulfonyl fluoride, 50 mM sodium fluoride, 10 mM β-glycerophosphate, and Complete™ protease inhibitor cocktail [Roche]). Lysates were gently sonicated on ice and clarified by centrifugation at 12,000× g for 5 min at 4°C [40]. Protein concentration of the supernatant was determined by the Bradford method (BioRad). A dsDNA probe containing the *ISG15* IFN-stimulated response element (ISRE) was generated from annealed complementary oligonucleotides containing 4-base 5′ overhangs (sense strand: 5′-GATCCTCGGGAAAGGGAAACCGAAACTGA-3′) by labeling with Klenow DNA polymerase in the presence of 1 mM each of dGTP, dTTP, dATP and 100 µCi of [α^32^P]dCTP (3,000 Ci/mmol). Unincorporated nucleotides were removed by passage through Micro Bio-Spin 6 chromatography columns (BioRad). Binding reactions were performed in a 25-µl reaction containing 10 mM HEPES-KOH (pH 7.5), 50 mM KCL, 1 mM EDTA, 0.1 mM DTT, 0.1% Triton X-100, 2.5% glycerol, 2 µg bovine serum albumin and 2 µg salmon testes DNA. ^32^P-labeled oligonucleotide probe (0.5 ng) was added to each binding mixture, and then incubated for 20 min at room temperature. Protein-DNA complexes were resolved by electrophoresis in non-denaturing 5% acrylamide gels run at 4°C in 0.5× TBE (1× TBE is 90 mM Tris, 88 mM boric acid and 2 mM EDTA). Following electrophoresis, gels were dried and processed by autoradiography.

### Immunoblotting

Cells (10^7^) were washed once in PBS and lysed in 200 sl of lysis buffer (50 mM Tris-HCl [pH 8.0], 150 mM NaCl, 1 mM EDTA, 1% Triton X-100, 1 mM sodium orthovanadate, 1 mM phenylmethylsulfonyl fluoride, 50 mM sodium fluoride, 10 mM β-glycerophosphate, and Complete™ protease inhibitor cocktail [Roche]) by incubation on ice for 10 min. Insoluble material was removed by centrifugation at 12,000× g for 10 min, and protein concentration of the supernatant determined by the Bradford method (BioRad). Fifty micrograms of protein was fractionated by SDS-PAGE, transferred to an Immobilon P membrane (Millipore), and immunoblotted using an enhanced chemiluminescence detection system (Amersham). For detection of STAT1 and STAT2 phosphorylation, blots were initially probed with a phosphorylation site-specific antibody, subsequently stripped of antibody in 62.5 mM Tris-HCl (pH 6.8), 100 mM 2-mercaptoethanol and 2% SDS (50°C for 30 min) and re-probed with a phosphorylation-state independent antibody. Immunoreactive proteins were detected with secondary antibodies conjugated to horseradish peroxidase. Blots were then stripped a second time and re-probed with a mouse monoclonal antibody to β-actin (Amersham) as a control for protein loading. Primary antibodies used for immunoblotting were rabbit polyclonal antisera to STAT1 p84/p91 (Santa Cruz Biotechnology), phospho-STAT1 (Tyr701 and Ser727; Upstate Biotechnology), STAT2 (Santa Cruz Biotechnology), phospho-STAT2 (Tyr689; Upstate Biotechnology), and IRF9/ISGF-3γ/p48 (Santa Cruz Biotechnology). Detection of FLAG-UBP43 was with anti-FLAG antibody (M2; Sigma). Signal intensity was quantified using ImageJ [41].

## Supporting Information

Figure S1 Duration of tyrosine phosphorylation of STAT1 is independent of EBV status. Two EBV-positive BL cell lines (KemI and MutuI) as well as two independently derived matched sets of EBV-negative (Ak− and 2A8−) and EBV-positive (Ak+ and 2A8+) Akata BL cells were treated with IFN-α for up to 24 h. Tyrosine phosphorylation of STAT1 was monitored by immunoblotting, as in Figs. 4 and 5. The faster-migrating background band (asterisk) served as protein loading controls on all blots.
(0.55 MB TIF)

## References

[1] Boxer LM, Dang CV (2001). Translocations involving c-myc and c-myc function.. Oncogene.

[2] Cai X, Schafer A, Lu S, Bilello JP, Desrosiers RC (2006). Epstein-Barr virus microRNAs are evolutionarily conserved and differentially expressed.. PLoS Pathog.

[3] Kieff ED, Rickinson AB, Knipe DM, H PM, Griffin DE, Lamb RA, Martin MA, Roizman B, Straus SE (2007). Epstein-Barr Virus and Its Replication.. Fields Virology. 5th ed.

[4] Pfeffer S, Zavolan M, Grasser FA, Chien M, Russo JJ (2004). Identification of Virus-Encoded MicroRNAs.. Science.

[5] Thorley-Lawson DA, Allday MJ (2008). The curious case of the tumour virus: 50 years of Burkitt's lymphoma.. Nat Rev Microbiol.

[6] Hochberg D, Middeldorp JM, Catalina M, Sullivan JL, Luzuriaga K (2004). Demonstration of the Burkitt's lymphoma Epstein-Barr virus phenotype in dividing latently infected memory cells in vivo.. Proc Natl Acad Sci U S A.

[7] Nanbo A, Inoue K, Adachi-Takasawa K, Takada K (2002). Epstein-Barr virus RNA confers resistance to interferon-alpha-induced apoptosis in Burkitt's lymphoma.. Embo J.

[8] Ruf IK, Lackey KA, Warudkar S, Sample JT (2005). Protection from Interferon-Induced Apoptosis by Epstein-Barr Virus Small RNAs Is Not Mediated by Inhibition of PKR.. J Virol.

[9] Samanta M, Iwakiri D, Kanda T, Imaizumi T, Takada K (2006). EB virus-encoded RNAs are recognized by RIG-I and activate signaling to induce type I IFN.. Embo J.

[10] Geiger TR, Martin JM (2006). The Epstein-Barr virus-encoded LMP-1 oncoprotein negatively affects Tyk2 phosphorylation and interferon signaling in human B cells.. J Virol.

[11] Malakhov MP, Malakhova OA, Kim KI, Ritchie KJ, Zhang DE (2002). UBP43 (USP18) specifically removes ISG15 from conjugated proteins.. J Biol Chem.

[12] Malakhova OA, Yan M, Malakhov MP, Yuan Y, Ritchie KJ (2003). Protein ISGylation modulates the JAK-STAT signaling pathway.. Genes Dev.

[13] Malakhova OA, Kim KI, Luo JK, Zou W, Kumar KG (2006). UBP43 is a novel regulator of interferon signaling independent of its ISG15 isopeptidase activity.. Embo J.

[14] Shimizu N, Tanabe-Tochikura A, Kuroiwa Y, Takada K (1994). Isolation of Epstein-Barr virus (EBV)-negative cell clones from the EBV-positive Burkitt's lymphoma (BL) line Akata: malignant phenotypes of BL cells are dependent on EBV.. J Virol.

[15] Komano J, Maruo S, Kurozumi K, Oda T, Takada K (1999). Oncogenic role of Epstein-Barr virus-encoded RNAs in Burkitt's lymphoma cell line Akata.. J Virol.

[16] Komano J, Sugiura M, Takada K (1998). Epstein-Barr virus contributes to the malignant phenotype and to apoptosis resistance in Burkitt's lymphoma cell line Akata.. J Virol.

[17] Ruf IK, Rhyne PW, Yang C, Cleveland JL, Sample JT (2000). Epstein-Barr virus small RNAs potentiate tumorigenicity of Burkitt lymphoma cells independently of an effect on apoptosis.. J Virol.

[18] Ruf IK, Rhyne PW, Yang H, Borza CM, Hutt-Fletcher LM (1999). Epstein-barr virus regulates c-MYC, apoptosis, and tumorigenicity in Burkitt lymphoma.. Mol Cell Biol.

[19] Kelly G, Bell A, Rickinson A (2002). Epstein-Barr virus-associated Burkitt lymphomagenesis selects for downregulation of the nuclear antigen EBNA2.. Nat Med.

[20] Yasukawa H, Sasaki A, Yoshimura A (2000). Negative regulation of cytokine signaling pathways.. Annu Rev Immunol.

[21] Wen Z, Zhong Z, Darnell JE (1995). Maximal activation of transcription by Stat1 and Stat3 requires both tyrosine and serine phosphorylation.. Cell.

[22] Der SD, Zhou A, Williams BR, Silverman RH (1998). Identification of genes differentially regulated by interferon alpha, beta, or gamma using oligonucleotide arrays.. Proc Natl Acad Sci U S A.

[23] Krebs DL, Hilton DJ (2001). SOCS proteins: negative regulators of cytokine signaling.. Stem Cells.

[24] Randall G, Chen L, Panis M, Fischer AK, Lindenbach BD (2006). Silencing of USP18 potentiates the antiviral activity of interferon against hepatitis C virus infection.. Gastroenterology.

[25] Loeb KR, Haas AL (1992). The interferon-inducible 15-kDa ubiquitin homolog conjugates to intracellular proteins.. J Biol Chem.

[26] Kim KI, Yan M, Malakhova O, Luo JK, Shen MF (2006). Ube1L and protein ISGylation are not essential for alpha/beta interferon signaling.. Mol Cell Biol.

[27] Knobeloch KP, Utermohlen O, Kisser A, Prinz M, Horak I (2005). Reexamination of the role of ubiquitin-like modifier ISG15 in the phenotype of UBP43-deficient mice.. Mol Cell Biol.

[28] Osiak A, Utermohlen O, Niendorf S, Horak I, Knobeloch KP (2005). ISG15, an interferon-stimulated ubiquitin-like protein, is not essential for STAT1 signaling and responses against vesicular stomatitis and lymphocytic choriomeningitis virus.. Mol Cell Biol.

[29] Kang D, Jiang H, Wu Q, Pestka S, Fisher PB (2001). Cloning and characterization of human ubiquitin-processing protease-43 from terminally differentiated human melanoma cells using a rapid subtraction hybridization protocol RaSH.. Gene.

[30] Li XL, Hassel BA (2001). Involvement of proteasomes in gene induction by interferon and double-stranded RNA.. Cytokine.

[31] Kim KI, Malakhova OA, Hoebe K, Yan M, Beutler B (2005). Enhanced antibacterial potential in UBP43-deficient mice against Salmonella typhimurium infection by up-regulating type I IFN signaling.. J Immunol.

[32] Ritchie KJ, Hahn CS, Kim KI, Yan M, Rosario D (2004). Role of ISG15 protease UBP43 (USP18) in innate immunity to viral infection.. Nat Med.

[33] Liu LQ, Ilaria R, Kingsley PD, Iwama A, van Etten RA (1999). A novel ubiquitin-specific protease, UBP43, cloned from leukemia fusion protein AML1-ETO-expressing mice, functions in hematopoietic cell differentiation.. Mol Cell Biol.

[34] Schwer H, Liu LQ, Zhou L, Little MT, Pan Z (2000). Cloning and characterization of a novel human ubiquitin-specific protease, a homologue of murine UBP43 (Usp18).. Genomics.

[35] Rempel LA, Austin KJ, Ritchie KJ, Yan M, Shen M (2007). Ubp43 gene expression is required for normal Isg15 expression and fetal development.. Reprod Biol Endocrinol.

[36] Ritchie KJ, Malakhov MP, Hetherington CJ, Zhou L, Little MT (2002). Dysregulation of protein modification by ISG15 results in brain cell injury.. Genes Dev.

[37] Yan M, Luo JK, Ritchie KJ, Sakai I, Takeuchi K (2007). Ubp43 regulates BCR-ABL leukemogenesis via the type 1 interferon receptor signaling.. Blood.

[38] Fok V, Friend K, Steitz JA (2006). Epstein-Barr virus noncoding RNAs are confined to the nucleus, whereas their partner, the human La protein, undergoes nucleocytoplasmic shuttling.. J Cell Biol.

[39] Sample J, Henson EB, Sample C (1992). The Epstein-Barr virus nuclear protein 1 promoter active in type I latency is autoregulated.. J Virol.

[40] Scholer HR, Hatzopoulos AK, Balling R, Suzuki N, Gruss P (1989). A family of octamer-specific proteins present during mouse embryogenesis: evidence for germline-specific expression of an Oct factor.. Embo J.

[41] Abramoff MD, Magelhaes PJ, Ram SJ (2004). Image Processing with ImageJ.. Biophotonics International.

