# Supplementary figures and images for: Epstein-Barr Virus Independent Dysregulation of UBP43 Expression Alters Interferon-Stimulated Gene Expression in Burkitt Lymphoma
Source: PLoS One. 2009 Jun 24;4(6):e6023. doi: 10.1371/journal.pone.0006023 (PMC2696598; doi:10.1371/journal.pone.0006023)

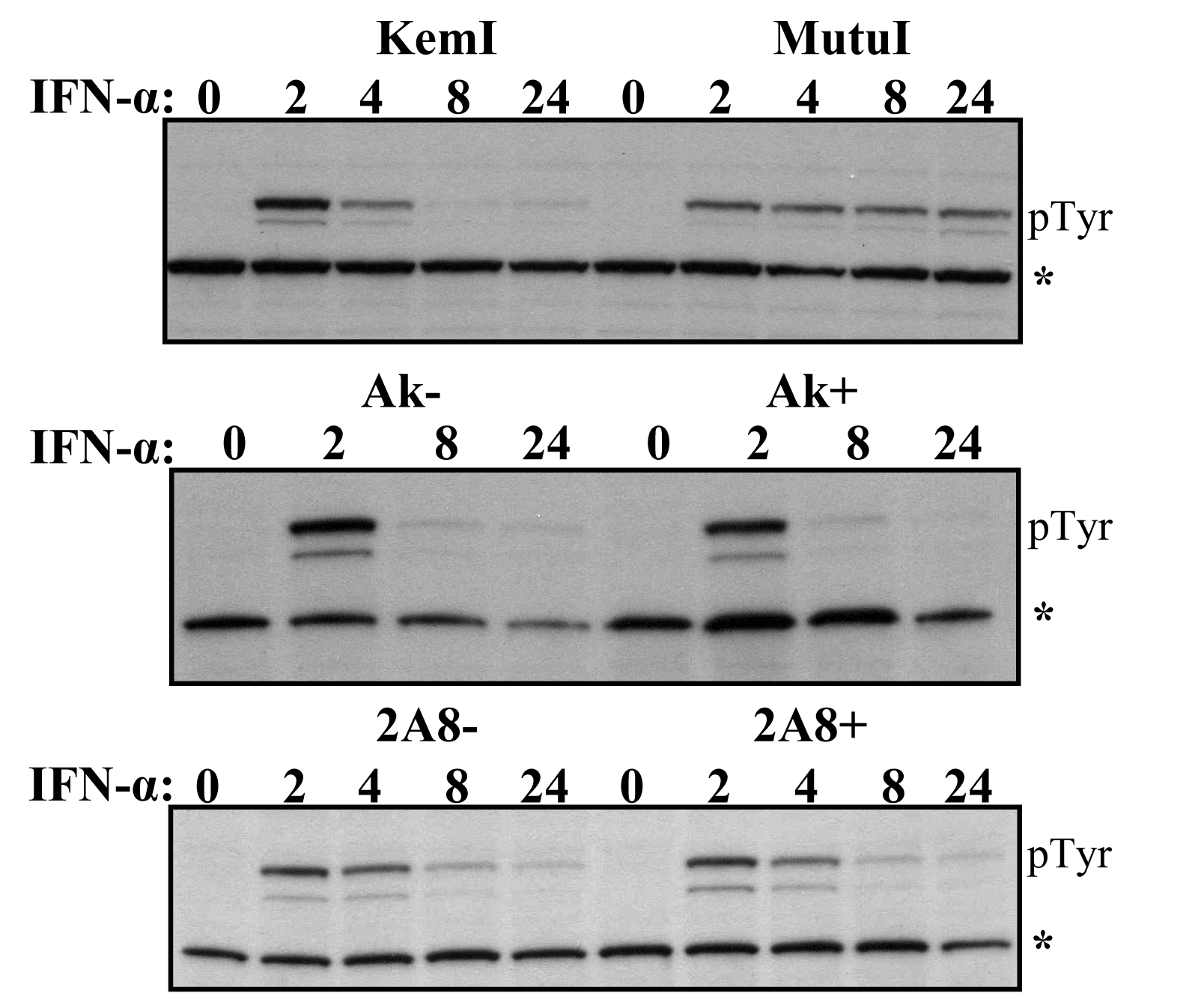

Supplement: Figure S1 — Duration of tyrosine phosphorylation of STAT1 is independent of EBV status. Two EBV-positive BL cell lines (KemI and MutuI) as well as two independently derived matched sets of EBV-negative (Ak− and 2A8−) and EBV-positive (Ak+ and 2A8+) Akata BL cells were treated with IFN-α for up to 24 h. Tyrosine phosphorylation of STAT1 was monitored by immunoblotting, as in Figs. 4 and 5. The faster-migrating background band (asterisk) served as protein loading controls on all blots. (0.55 MB TIF) [file pone.0006023.s001.tif]
